# Identification and Functional Analysis of GTP Cyclohydrolase II in *Candida glabrata* in Response to Nitrosative Stress

**DOI:** 10.3389/fmicb.2022.825121

**Published:** 2022-03-02

**Authors:** Ryo Nasuno, Soma Suzuki, Sayoko Oiki, Daisuke Hagiwara, Hiroshi Takagi

**Affiliations:** ^1^Division of Biological Science, Graduate School of Science and Technology, Nara Institute of Science and Technology, Ikoma, Japan; ^2^Faculty of Life and Environmental Sciences, University of Tsukuba, Tsukuba, Japan; ^3^Microbiology Research Center for Sustainability, University of Tsukuba, Tsukuba, Japan

**Keywords:** *Candida glabrata*, nitric oxide, GTP cyclohydrolase II, riboflavin, macrophage, silkworm

## Abstract

Reactive nitrogen species (RNS) are signal molecules involved in various biological events; however, excess levels of RNS cause nitrosative stress, leading to cell death and/or cellular dysfunction. During the process of infection, pathogens are exposed to nitrosative stress induced by host-derived RNS. Therefore, the nitrosative stress resistance mechanisms of pathogenic microorganisms are important for their infection and pathogenicity, and could be promising targets for antibiotics. Previously, we demonstrated that the *RIB1* gene encoding GTP cyclohydrolase II (GCH2), which catalyzes the first step of the riboflavin biosynthesis pathway, is important for nitrosative stress resistance in the yeast *Saccharomyces cerevisiae*. Here, we identified and characterized the *RIB1* gene in the opportunistic pathogenic yeast *Candida glabrata*. Our genetic and biochemical analyses indicated that the open reading frame of CAGL0F04279g functions as *RIB1* in *C. glabrata* (*CgRIB1*). Subsequently, we analyzed the effect of *CgRIB1* on nitrosative stress resistance by a growth test in the presence of RNS. Overexpression or deletion of *CgRIB1* increased or decreased the nitrosative stress resistance of *C. glabrata*, respectively, indicating that GCH2 confers nitrosative stress resistance on yeast cells. Moreover, we showed that the proliferation of *C. glabrata* in cultures of macrophage-like cells required the GCH2-dependent nitrosative stress detoxifying mechanism. Additionally, an infection assay using silkworms as model host organisms indicated that *CgRIB1* is indispensable for the virulence of *C. glabrata*. Our findings suggest that the GCH2-dependent nitrosative stress detoxifying mechanism is a promising target for the development of novel antibiotics.

## Introduction

Reactive nitrogen species (RNS) including nitric oxide (NO) function as ubiquitous signal molecules in a variety of biological phenomena in many kinds of organisms including mammals, plants, and microorganisms (Pacher et al., 2007; Wink et al., 2011). For example, NO regulates the relaxation of vascular smooth muscle cells and neurotransmission in mammals (Ferrer-Sueta et al., 2018). We previously reported that NO conferred high-temperature stress resistance on cells of the yeast *Saccharomyces cerevisiae* (Nishimura et al., 2013; Nasuno et al., 2014). NO exerts its functions by the activation of soluble guanylate cyclase (sGC) through its binding to the heme of sGC in mammals (Francis et al., 2010; Hunt and Lehnert, 2015). NO also mediates post-translational modification, such as *S*-nitrosylation and oxidation of cysteine residues on proteins to induces certain biological activities (Bai et al., 2011; Nakato et al., 2015). The NO_2_ radical, one of the most unstable RNS derived from NO, induces the nitration of tyrosine residues on proteins, leading to loss, change or even gain of function (Kang et al., 2015; Porter et al., 2020).

In contrast to the physiological roles of RNS as signals described above, excess concentrations of RNS are toxic and cause nitrosative stress, leading to cellular dysfunction and/or cell death (Boscá and Hortelano, 1999; Pacher et al., 2007; Yoshikawa et al., 2016). Animal defense systems use nitrosative stress induced by RNS as a weapon to kill pathogens. Macrophages activated during infection release NO, and then NO and NO-derived RNS like peroxynitrite attack pathogenic microorganisms (von Bargen et al., 2011). Therefore, the nitrosative stress detoxifying mechanisms in pathogens are important for their infectivity and/or pathogenicity. A previous study showed that one of the important NO-degrading enzymes, NO reductase, is involved in the virulence of *Pseudomonas aeruginosa*, by an infection assay using the silkworm *Bombyx mori* as a model host organism (Arai and Iiyama, 2013). Flavohemoglobin (fHb) is another NO detoxification system, which degrades NO oxidatively or reductively under aerobic or anaerobic conditions, respectively (Gardner et al., 1998; Kim et al., 1999). A previous report using the pathogenic fungus *Candida albicans* indicated that the survival of mice infected with a *C. albicans* strain lacking fHb is significantly higher than that infected with the wild-type strain, suggesting that the fHb deficiency reduces the virulence of *C. albicans* (Hromatka et al., 2005).

Recently, we discovered a novel nitrosative stress resistance system dependent on GTP cyclohydrolase II (GCH2) in the yeast *S. cerevisiae* (Anam et al., 2020). GCH2, the first-step and rate-limiting enzyme of the riboflavin (RF) biosynthesis pathway, converts its substrate GTP into 2,5-diamino-6-(5-phospo-d-ribosylamino)-pyrimidin-4(3H)-one (DARP); then DARP scavenges NO, leading to nitrosative stress resistance. The *RIB1* gene encoding GCH2 is conserved among a wide range of microorganisms including bacteria, yeasts, and fungi, but not in mammals. Therefore, GCH2 would be a promising target protein for development of novel antibiotics.

The asexual and haploid yeast *Candida glabrata* is an opportunistic pathogen causing candidiasis especially in patients with immunocompromised state, with diabetes or cancer, treated with antimicrobials, and using the medical devices in surgery like catheters (Malani et al., 2005; Antinori et al., 2016). After *C. albicans*, *C. glabrata* is the second most frequently isolated *Candida* species causing invasive candidiasis (Hassan et al., 2021). Although *C. glabrata* is less virulent than other *Candida* species, such as *C. albicans*, it causes high mortality when the immune systems of patients are suppressed by underlying diseases like acquired immune deficiency syndrome (AIDS), treatment with immunosuppressive drugs, or aging (Hassan et al., 2021). *Candida* species including *C. glabrata* cause catheter-related bloodstream infections, which is a life-threatening complication, in the patients using catheters for parenteral nutrition (Phua et al., 2019). *C. glabrata* has unique characteristics especially during infection. Several cytokines are produced when organisms are infected with pathogenic microorganisms like *C. albicans* or *Mycobacterium tuberculosis*, leading to the activation of macrophages and maturation of phagolysosomes (Lionakis and Netea, 2013). When macrophages come into contact with pathogenic microbes, various saccharides including mannan, glucan, key structural components of fungi and yeasts, and lipopolysaccharide (LPS), a key component of bacterial membranes, bind to the specific receptors on the macrophage, activating it (Lionakis and Netea, 2013). The cytokine production induced by infection with *C. glabrata* is clearly lower than that by *C. albicans* (Seider et al., 2011). Whereas, the infection with *C. glabrata* does not cause NO production from macrophages unlike *C. albicans* (Kaur et al., 2007), even though human osteoblasts infected with *C. glabrata* produce NO (Muñoz-Duarte et al., 2016). Additionally, an *in vitro* infection assay demonstrated that *C. glabrata* was much less toxic to macrophages than *C. albicans*, despite the fact that *C. glabrata* was more effectively phagocytosed by macrophages than *C. albicans* (Dementhon et al., 2012). Further studies indicated that *C. glabrata* could even replicate inside macrophage cells (Seider et al., 2011). Importantly, *C. glabrata* is relatively resistant to azole type antifungals, the most widely used class of antifungal drugs, due to its energy-dependent efflux system (Parkinson et al., 1995). Therefore, it is highly desirable to discover novel antifungals that would be effective against *C. glabrata*, and/or a feasible drug target for that species.

Here, we identified the gene encoding GCH2 and characterized the GCH2-dependent NO resistance system in *C. glabrata*. Furthermore, we demonstrated that the NO detoxifying mechanism mediated by GCH2 was essential for the virulence of *C. glabrata* with infection assays using macrophage cultures and silkworms.

## Materials and Methods

### Strains, Plasmids, and Medium

*Candida glabrata* KUE100-1 strain with a CBS138 background (Ueno et al., 2007) was used as a wild-type (WT) strain. The strain lacking CAGL0F04279g was constructed as follows. The DNA fragment from 500 bp upstream to 500 bp downstream of CAGL0F04279g was introduced to a plasmid pUC18 by PCR and InFusion reaction (Clontech, Mountain View, CA, United States) using pUC18 and the genomic DNA of WT strain as templates and primers listed in Supplementary Table 1. The resultant plasmid pUC18-CAGL0F04279g was linearized to remove the open reading frame (ORF) of CAGL0F04279g by the inverse PCR and then conjugated to a *natNT2* transformation marker from pFA6a-natNT2, generating the plasmid pUC18-ΔCAGL0F04279g-natNT2. The DNA fragment consisting of 500 bp upstream region of CAGL0F04279g, a natNT2 transformation marker, and 500 bp downstream region of CAGL0F04279g was amplified by PCR using pUC18-ΔCAGL0F04279g-natNT2 as a template and the primers listed in Supplementary Table 1, and then introduced to WT cells for homologous recombination. The conventional colony-direct PCR was performed to analyze the transformants. The clones showing the genotype of recombination were identified as the *rib1*Δ strain, as described in detail in the results section. Subsequently, a plasmid pUC18-CAGL0F04279g was linearized to remove the ORF of CAGL0F04279g by the inverse PCR and then conjugated to a *kanMX6* transformation marker from pFA6a-kanMX6, to generate the plasmid pUC18-ΔCAGL0F04279g-kanMX6 harboring the 500 bp upstream region of CAGL0F04279g, a *kanMX6* transformation marker, and 500 bp downstream region of CAGL0F04279g, tandemly. The *rib1*Δ strain was transformed with the DNA fragment consisting of the DNA sequence of 500 bp upstream of CAGL0F04279g, a *kanMX6* transformation marker, and 500 bp downstream of CAGL0F04279g amplified from pUC18-ΔCAGL0F04279g-kanMX6. The resultant transformants were analyzed by the conventional colony-direct PCR analysis. In order to delete *YHB1*, pUC18-YHB1-natNT2 harboring the 500 bp upstream region of *YHB1*, a *natNT2* transformation marker, and 500 bp downstream region of *YHB1*, tandemly, was constructed using the primers listed in Supplementary Table 1, pUC18, and pFA6a-natNT2 in the similar way to the construction of pUC18-ΔCAGL0F04279g-natNT2. The DNA fragment consisting of the DNA sequence of 500 bp upstream of *YHB1*, a *natNT2* transformation marker, and 500 bp downstream of *YHB1* amplified from pUC18-YHB1-natNT2 was introduced to WT cells.

A centromere-type plasmid pRS313 (ATCC) was introduced to complement the histidine auxotrophy of *C. glabrata* used in this study. A centromere-type plasmid pCU-PDC1 was used for gene expression in *C glabrata* (Zordan et al., 2013). The coding region including epitope tag in pET53-CAGL0F04279g, which was constructed as described below, was amplified by PCR using the primers listed in Supplementary Table 1, and then introduced into pCU-PDC1, generating pCU-PDC1-CAGL0F04279g to express CAGL0F04279g under the control of the *PDC1* promoter. The plasmid pCU-PDC1-CAGL0F04279g was also used to overexpress CAGL0F04279g in the WT strain. In order to express *RIB1* from *S. cerevisiae*, the previously constructed plasmid pET53-RIB1 (Anam et al., 2020) was used as a template, followed by the same manipulation as the case of CAGL0F04279g.

*Candida glabrata* strains were cultured in a nutrient-rich medium YPD (1% yeast extract, 2% peptone, and 2% glucose) or a synthetic medium SD (2% glucose, 0.5% ammonium sulfate, 0.17% yeast nitrogen base without ammonium sulfate and amino acids) with pH 5.5 at 37°C in the presence of 50 μM RF, 200 μg/mL G418, 200 μg/mL nourseothricin, or 2% agar if necessary.

The primers, yeast strains, and plasmids used in this study were listed in Supplementary Tables 1–3, respectively.

### Quantitative PCR to Determine the Copy Number of CAGL0F04279g

The ORF of the *ACT1* gene was amplified from the genomic DNA of the WT and then inserted into the plasmid pUC18-CAGL0F04279g by PCR using the primer listed in Supplementary Table 1 and InFusion reaction, generating the plasmid pUC18-CAGL0F04279g-*ACT1*. The genomic DNA of each strain of *C. glabrata* was extracted using Dr. GenTLE™ (from Yeast) High Recovery (TaKaRa Bio, Shiga, Japan) following the manufacturer’s instruction. A quantitative PCR with Real-Time PCR System QuantStudio 3 (Qiagen, Hilden, Germany) and SsoAdvanced Universal SYBR Green Supermix (Bio-Rad, Hercules, CA, United States), using the genomic DNA as a template and primers listed in Supplementary Table 1, was performed in order to determine the copy number of CAGL0F04279g in each strain. The copy number of CAGL0F04279g and *ACT1* were calculated from each *Ct* value using the standard curve using the plasmid pUC18-CAGL0F04279g-*ACT1* as a template. The obtained copy number of CAGL0F04279g was normalized by that of *ACT1*.

### Phenotypic Analysis of *Candida glabrata* on Solid Medium

Yeast cells were cultured in SD medium containing 50 μM RF until exponential growth phase. The serial dilutions were spotted on to SD solid medium with pH 5.5 and then cultured at 37°C. Fifty μM RF or the indicated concentration of sodium nitrite was added to solid medium to analyze the RF auxotrophy or nitrosative stress resistance, respectively.

### Analysis of GTP Cyclohydrolase II Enzymatic Activity

The CAGL0F04279g ORF was cloned into a plasmid pET53 by BP and LR reactions in Gateway technology (Invitrogen, Carlsbad, CA, United States) and PCR with the primers listed in Supplementary Table 1. The resultant plasmid pET53-CAGL0F04279g was introduced into *Escherichia coli* BL21 (DE3) strain. The transformant cells were cultured in LB medium with 100 μg/mL ampicillin until 0.8 of OD_600_ and then 0.1 mM isopropyl-β-D-thiogalactopyranoside was added, followed by further incubation at 30°C for 16 h. The harvested cells were washed with and suspended in 20 mM sodium phosphate (pH 7.4), 500 mM NaCl (Buffer A) and then disrupted by sonication. The supernatant after centrifugation was subjected to purification using Ni Sepharose 6 Fast Flow (Cytiva, Tokyo, Japan). Eluate with Buffer A containing 500 mM imidazole was dialyzed against 50 mM Tris–HCl (pH 8.0), 1 mM TCEP, 10% glycerol and then the sample containing the recombinant protein from CAGL0F04279g was used further analyses. The purity was analyzed by SDS-PAGE and CBB staining.

The recombinant enzyme was incubated in the mixture containing 100 mM Tris–HCl (pH 8.8), 5 mM MgCl_2_, 2.5 mM dithiothreitol, and 1 mM GTP at 37°C for 30 min. Fifty μL of the stop solution containing 1% diacetyl and 15% trichloroacetic acid was added and then incubated at 37°C for 70 min. The fluorescence intensity of the supernatant after centrifugation was measured using a microplate reader TriStar LB942 (Berthold Technologies, Bad Wildbad, Germany) with an excitation filter F355 and emission filter F460. The standard curve of 6,7-dimethylpterin was drawn to quantify generated DARP. One unit of the specific activity of GTPCH2 was defined as the amount of enzyme to produce 1 μmol of DARP per min, which was calculated using the value at 15 min incubation.

Yeast cells cultured in SD with RF for 24 h were resuspended in 20 mM sodium phosphate buffer (pH 7.4) containing 0.5 M NaCl, followed by cell disruption using Multi-beads shocker (Yasui Kikai, Osaka, Japan) with glass beads. The supernatant after centrifuge was collected and then the GCH2 activity in it was analyzed as described above.

### *In vitro* Nitric Oxide Quenching Assay

The recombinant protein expressed from CAGL0F04279g was incubated for 30 min at 37°C with or without GTP in the same condition as the GCH2 activity assay described above. Subsequently, 7 μM 4-amino-5-methylamino-2′;,7′-difluorofluorescein (DAF-FM) and 400 μM 1-hydroxy-2-oxo-3-(3-aminopropyl)-3-isopropyl-1-triazene (NOC-5) was added and then further incubated at room temperature and the fluorescence intensity was monitored to estimate the remaining NO in solution over time using a microplate reader TriStar LB942 (Berthold Technologies, Bad Wildbad, Germany) with an excitation filter F485 and emission filter F535.

### Culture of Macrophage-Like Cells

Dulbecco’s modified Eagle medium (DMEM) with 4.5 mg/L glucose, 4 mM L-glutamine, 15 mg/L phenol red, and 25 mM HEPES (FUJIFILM Wako Pure Chemical, Osaka, Japan) supplemented with 1/100 volume of Antibiotic-Antimycotic Mixed Stock Solution (100×) (Stabilized) (Nacalai Tesque, Kyoto, Japan), 1/100 volume of MEM Non-Essential Amino Acids Solution (100×) (Nacalai Tesque), and 1/10 volume of Fetal Bovine Serum, French (FBS, French) (MP Biomedicals, Irvine, CA, United States) was used as DMEM-A in this study. DMEM with 4.5 mg/L glucose, 4 mM L-glutamine, and 25 mM HEPES (FUJIFILM Wako Pure Chemical, Osaka, Japan) supplemented with 1/10 volume of FBS, French was also used as DMEM-B.

Macrophage-like cells RAW264.7 (ATCC) were cultured in DMEM-A at 37°C with 5% CO_2_. After 3 passages, cells were resuspended in DMEM-B and then 2.5 × 10^4^ cells adhered to a polystyrene cell culture plate were further cultured for 24 h in the presence of 100 ng/mL LPS, 100 μM NOS inhibitor *N^G^*-nitro-L-arginine methyl ester (NAME), or 100 μg/mL RF if necessary. The supernatant of RAW264.7 cell culture in DMEM-B was reacted with 2.5 μM DAF-FM and then the fluorescence was measured using a microplate reader TriStar LB942 (Berthold Technologies, Bad Wildbad, Germany) with an excitation filter F485 and emission filter F535.

Each strain of *C. glabrata* cultured in SD medium with RF was washed and then 1.2 × 10^5^ cells of yeast were added to 2.5 × 10^4^ cells of RAW264.7, which were cultured in DMEM-A and resuspended in DMEM-B as described above, followed by further culture in DMEM-B for 2 h in the presence of LPS, NAME, and RF if necessary. Subsequently, RAW264.7 cells were washed with 1 mL of PBS twice and cultured in DMEM-B for the indicated time in the presence of LPS, NAME, and RF if necessary. After removal of medium, macrophage-like cells were incubated in 0.1% Triton X-100 for 1 min for cell lysis and the supernatant was spread onto YPD plate. The number of colonies generated after a few days culture at 37°C were counted to estimate the viable cell number of yeast inside macrophage.

### Silkworm Infection Assay

*Candida glabrata* strains were cultured in SC-Ura-His medium containing 50 μM RF (WT, *rib1*ΔΔ, *yhb1*Δ) at 37°C for 2 days. Cells were harvested and resuspended in 0.05% Tween 20/0.6% NaCl at 2 × 10^8^/mL. Red food coloring agent was added to the suspensions.

Four-molt silkworms and SILKMATE 2S were purchased from Ehime Sansyu (Ehime, Japan). Silkworms (the first day of fifth-instar larvae) were fed with SILKMATE 2S at 25°C for 2 days, followed by fasting for a day. The larvae were injected with 50 μL of fungal suspensions into intra-hemolymph using a 29-gage needle. After infection, the larvae were maintained without food at 30°C and survival percent was monitored for 4 days. Twelve silkworms were used for each strain. Statistical analyses of the survival percent were performed using the Kaplan-Meier method (Cochran–Mantel–Haenszel).

## Results

### *Candida glabrata* Harbors Two Copies of Putative *RIB1* Gene CAGL0F04279g

A previous study determined the genomic DNA sequence of the *C. glabrata* CBS138 strain, indicating that the ORF of CAGL0F04279g in *C. glabrata* is homologous to the *RIB1* gene in *S. cerevisiae* (Dujon et al., 2004). Our BLAST search using an expected amino acid sequence translated from CAGL0F04279g, whose length is 296 amino acids, indicated that the protein encoded by CAGL0F04279g exhibited 79.6% of identity and 1.0 × 10^–177^ of *e*-value against GCH2 encoded by *RIB1* gene in *S. cerevisiae*, which consists of 345 amino acids. Therefore, we hypothesized that the ORF of CAGL0F04279g functions as the *RIB1* gene encoding GCH2 in *C. glabrata* and then analyzed a *C. glabrata* strain lacking CAGL0F04279g.

**FIGURE 1:**
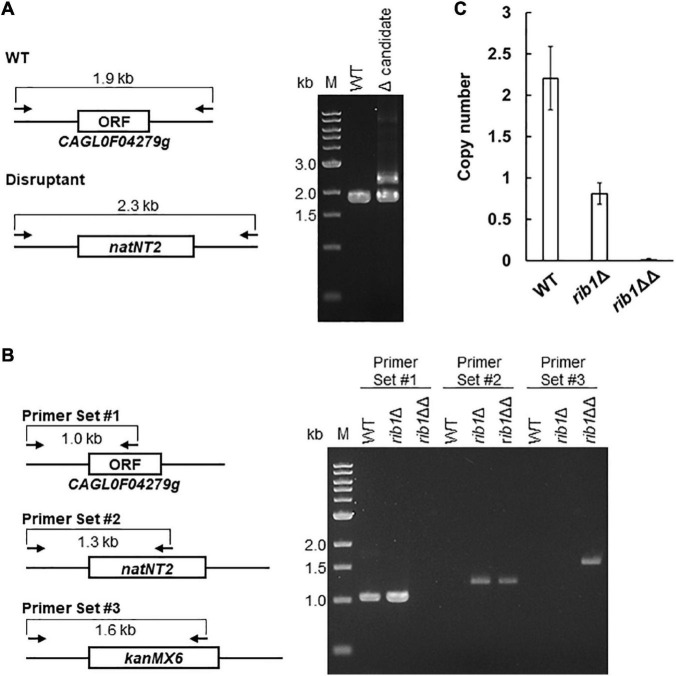
Deletion and copy number determination of CAGL0F04279g. **(A)** The result of PCR analysis of the transformants generated by the homologous recombination with a *natNT2* marker using the WT rain as a host is shown, with the expected genetic structure in the locus of CAGL0F04279g in WT and disruptant strains. Each arrow indicates the primers for PCR analysis. The expected length of amplified DNA fragment is indicated. **(B)** The result of PCR analysis of the candidate clones generated by the additional transformation with a *kanMX6* marker was shown, with the expected genetic structure in the locus of CAGL0F04279g in WT and disruptant. Each arrow indicates the primers for PCR analysis. The expected length of amplified DNA fragment was indicated. **(C)** Quantitative PCR analysis to determine the copy number of CAGL0F04279g. The copy number of CAGL0F04279g calculated using a standard curve was normalized to the copy number of *ACT1*. The values are the means and standard deviations of three independent experiments.

First, we deleted CAGL0F04279g by homologous recombination with a *natNT2* transformation marker using a WT strain as a host. The resultant transformants were analyzed by conventional PCR. Surprisingly, all candidate clones of the CAGL0F04279g disruptants exhibited not only the genotype of recombination with a *natNT2* marker but also the same genotype as the WT strain (Figure 1A). These results suggest that *C. glabrata* CBS138 strain possesses two or more copies of CAGL0F04279g, even though the previous genomic analysis suggested that there is only one copy of CAGL0F04279g. Subsequently, we deleted an additional copy of CAGL0F04279g using the clones showing both WT and disruptant genotypes (*rib1*Δ) as a host strain by a homologous recombination using a *kanMX6* transformation marker. As a result of PCR analysis, the resultant transformants (*rib1*ΔΔ) exhibited both the genotype of recombination with *natNT2* and that with *kanMX6*, indicating that the ORF of CAGL0F04279g no longer existed in the genome of the *rib1*ΔΔ strain (Figure 1B). We further measured the copy number of CAGL0F04279g by a quantitative PCR. An absolute quantification of the copy numbers of CAGL0F04279g with a standard curve, which was drawn with the plasmid harboring CAGL0F04279g using *ACT1* as a reference gene, demonstrated that the WT strain possessed two copies of CAGL0F04279g; in contrast, the *rib1*Δ strain possessed only one copy of CAGL0F04279g (Figure 1C). Next, we determined whether the DNA sequences of the two copies of CAGL0F04279g were the same or different. A DNA fragment from 509 bp upstream to 504 bp downstream of the ORF of CAGL0F04279g was cloned into plasmid pUC18. The independent clones of resultant plasmids were subjected to DNA sequencing analysis, and 8 independent clones exhibited the exact same DNA sequences. This suggests that the two copies of CAGL0F04279g in *C. glabrata* harbor the same DNA sequence.

The finding that *C. glabrata* harbors two copies of CAGL0F04279g in its genome is inconsistent with a previous report (Dujon et al., 2004). For further analyses in the present study, the *rib1*ΔΔ strain, from which both copies of CAGL0F04279g were deleted, was used as a CAGL0F04279g disruptant. The *rib1*Δ strain, from which one copy of CAGL0F04279g was deleted, was also used as a single-copy disruptant in additional experiments described below.

### CAGL0F04279g Functions as the *RIB1* Gene Encoding GTP Cyclohydrolase II in *Candida glabrata*

**FIGURE 2:**
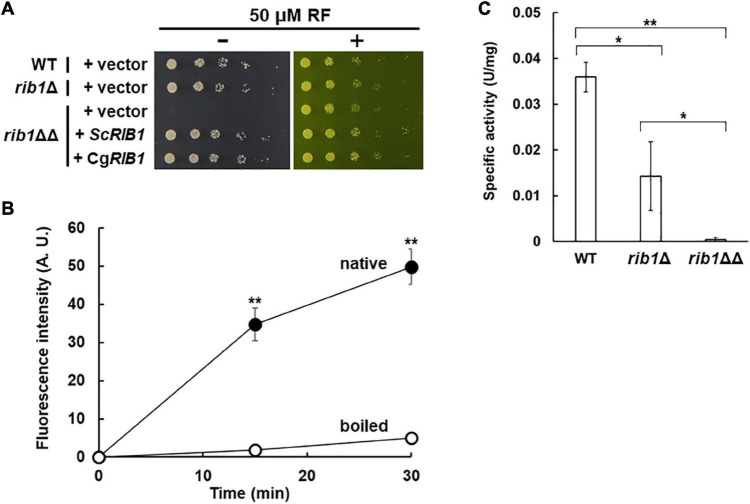
Identification of *CgRIB1* encoding GCH2 in *C. glabrata*. **(A)** Yeast cells were cultured in SD medium until exponential phase. Serial dilutions of each strain were spotted onto SD medium with pH 5.5 in the presence or absence of RF and then further cultured. **(B)** The protein expressed from CAGL0F04279g in *E. coli* was purified. The purified protein was incubated in the reaction mixture over time, with or without pre-boiling. The resultant solution was reacted with diacetyl and then the fluorescence was measured. The fluorescence at time of 0 min was used as a blank in each sample. **(C)** Yeast cell-free extract prepared from cells cultured in SD medium for 24 h was used as a crude enzyme. The values in panels **(B,C)** are the means and standard deviations of three independent experiments. *p < 0.05; **p < 0.001 by Student’s *t*-test.

The RF auxotrophic phenotypes of the WT, *rib1*Δ, and *rib1*ΔΔ strains were analyzed (Figure 2A). All strains grew at almost the same growth rate on a medium containing RF. However, in the absence of RF, the *rib1*ΔΔ strain did not grow while the WT and *rib1*Δ strains grew at the same rate. Importantly, the growth defect of the *rib1*ΔΔ strain was compensated for by the expression of the *RIB1* gene from *S. cerevisiae* (*ScRIB1*) or CAGL0F04279g. Subsequently, the enzymatic activity of the gene product of CAGL0F04279g was analyzed using the recombinant protein purified from *E. coli* cells expressing CAGL0F04279g by monitoring fluorescence derived from its reaction product (Figure 2B). When the native recombinant enzyme was included in the reaction mixture, the fluorescence increased in a time-dependent manner. On the other hand, the pre-boiled enzyme did not show increased fluorescence. These findings indicate that the recombinant protein exhibits GCH2 activity, which was calculated as 1.25 U/mg. Therefore, we concluded that CAGL0F04279g functions as *RIB1* encoding GCH2 in *C. glabrata* (*CgRIB1*).

Additionally, the GCH2 activity in yeast cell-free lysate was evaluated (Figure 2C). The WT and *rib1*Δ strains showed clear GCH2 activity, but the activity in the *rib1*Δ strain was almost 40% of that of the WT. Interestingly, there was no detectable GCH2 activity in the lysate from the *rib1*ΔΔ strain. These results suggest that the two copies of *CgRIB1* function to fully express active GCH2 almost equally, which is not surprising because the DNA sequences of the two copies of *CgRIB1* were identical as shown above.

### *RIB1* Is Essential for Nitrosative Stress Resistance in *Candida glabrata*

**FIGURE 3:**
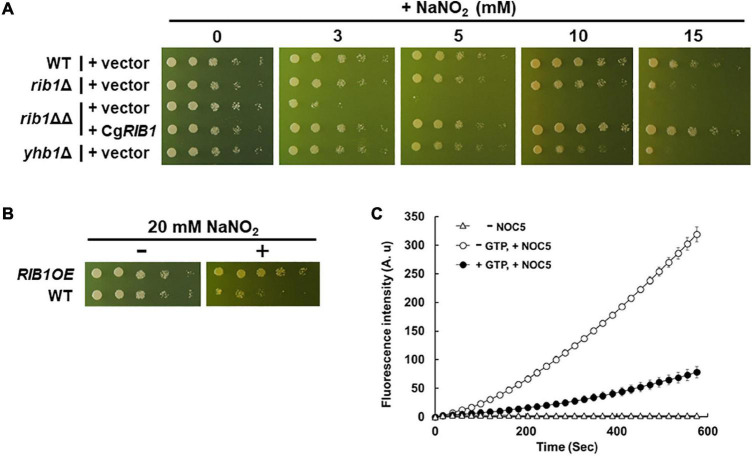
Nitrosative stress resistance mechanism mediated by *CgRIB1* in *C. glabrata*. **(A,B)** Yeast cells cultured in SD medium containing RF until exponential phase were spotted onto the SD medium with pH 5.5 containing RF with or without various concentration of sodium nitrite, followed by further cultivation. **(C)** The enzymatic reaction mixture using the recombinant CgGCH2 protein with or without its substrate GTP was mixed with DAF-FM, followed by the addition of NOC-5. The fluorescence intensity was monitored over time. The fluorescence intensity at the time of 0 min was used as a blank in each sample. The mixture of reaction buffer with DAF-FM lacking NOC-5 was used as a negative control. The values are the means and standard deviations of three independent experiments.

A previous study reported that the presence of nitrite induced nitrosative stress under acidic conditions (Zhou et al., 2013). Therefore, we evaluated the growth of *C. glabrata* on minimal medium containing sodium nitrite at pH 5.5 (Figure 3A). The growth of the WT strain decreased in a nitrite dose-dependent manner while the strain lacking the *YHB1* gene encoding fHb exhibited a more severe growth defect than the WT strain at a high concentration of nitrite. These results indicate that nitrosative stress induced by nitrite is toxic to *C. glabrata* under the conditions tested in this study. The growth of the *rib1*ΔΔ strain on medium containing nitrite was dramatically inhibited, which was compensated for by the plasmid carrying the *CgRIB1* gene. Indeed, overexpression of *CgRIB1* increased the growth of *C. glabrata* cells in the presence of nitrite (Figure 3B). These results indicate that *CgRIB1* is critical for nitrosative stress resistance in *C. glabrata*. Furthermore, we measured the NO-quenching activity of the reaction product of GCH2 encoded by *CgRIB1* (CgGCH2) using a fluorescent NO probe DAF-FM and an NO donor NOC-5 (Figure 3C). The time-dependent increase in fluorescence by the addition of NOC-5 was clearly inhibited by the enzymatic reaction mixture of CgGCH2; this effect was canceled out by the omission of GTP from the reaction mixture, suggesting that the product of CgGCH2 enzymatic reaction scavenges NO before it reacts with DAF-FM. These results indicate that *CgRIB1* confers nitrosative stress resistance on *C. glabrata* cells in the same way as in *S. cerevisiae* cells. Interestingly, the growth of the *rib1*Δ strain was also impaired by exposure to nitrite; however, the degree of its growth defect was milder than in the *rib1*ΔΔ strain (Figure 3A). This finding indicates that the effect of nitrosative stress depends on the copy number of *CgRIB1*.

### *RIB1*-Dependent Nitrosative Stress Resistance Is Important for Proliferation of *Candida glabrata* in Macrophage-Like Cells

**FIGURE 4:**
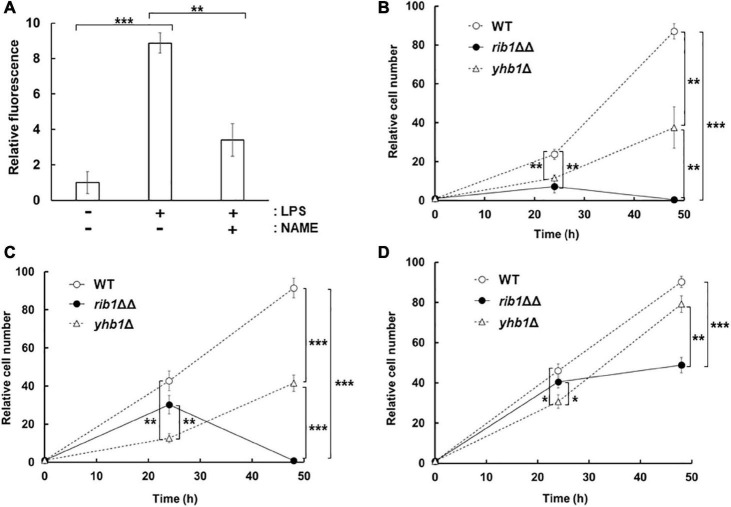
Proliferation of *C. glabrata* in macrophage-like cell. **(A)** NO production from RAW264.7 cells activated by LPS. The supernatant of cells incubated with LPS and/or NAME was reacted with DAF-FM and then the fluorescence intensity was measured. The fluorescence intensity before the incubation was used as a blank. **(B–D)** RAW264.7 cells infected with *C. glabrata* were washed and resuspended in DMEM-B, followed by further incubation for 24 or 48 h. After incubation, macrophage-like cells were lysed and the extracted solution were plated onto YPD with RF solid medium. Colonies formed after further cultivation for a few days at 37°C were counted. Relative cell number using that at 0 h as 1 was shown. The macrophage-like cells were pretreated with LPS **(B)**, LPS and RF **(C)**, or LPS, RF, and NAME **(D)**, respectively, and then further incubated in the presence of these additives. The values are the means and standard deviations of three independent experiments. *p < 0.05; **p < 0.01; ***p < 0.001 by Student’s *t*-test.

In order to examine the effect of CgGCH2 on the virulence of *C. glabrata*, we evaluated the replication of yeast in a culture of RAW264.7 cells, a model of activated macrophages. At first, NO production was induced from RAW264.7 cells by LPS treatment using DAF-FM (Figure 4A). The fluorescence derived from NO was increased when RAW264.7 cells were treated with LPS, which was clearly suppressed by the additional treatment with the NOS inhibitor NAME. These results showed that the LPS treatment and NAME addition performed in this study efficiently induced and inhibited NO production from RAW264.7 cells, respectively. Subsequently, *C. glabrata* cells were incorporated into the culture of LPS-treated RAW264.7 cells, and the viable cell numbers of yeast were analyzed (Figure 4B). The viable cell number of the WT strain increased over time. The strain lacking *YHB1* grew in the RAW264.7 cells culture more slowly than the WT strain, probably due to nitrosative stress induced by the treatment with LPS. The cell number of the *rib1*ΔΔ strain increased slightly until 24 h incubation; however, it decreased during the next 24 h. In order to exclude the effect of RF auxotrophy on the phenotype of the *rib1*ΔΔ strain, the macrophage infection assay was performed in the presence of 100 μg/mL RF, which has been reported to increase the RF concentration inside macrophage cells to 100 μM (Dey and Bishayi, 2016; Figure 4C). The result showed that for the first 24 h, the *rib1*ΔΔ strain grew better in the presence than in the absence of RF; however, its viable cell number dropped in the following 24-h incubation. When the mammalian NOS inhibitor NAME was added to evaluate the inhibitory effect of NO produced from RAW264.7 cells on the growth of yeast (Figure 4D), the growth of *yhb1*Δ cells was recovered to almost the same level as WT cells, suggesting that the growth defect of *yhb1*Δ cells had been due to nitrosative stress. Importantly, the decrease in cell number of the *rib1*ΔΔ strain during the second 24 h incubation was dramatically rescued by treatment with NAME, which indicates that the severe growth defect of the *rib1*ΔΔ strain was also induced by nitrosative stress.

### *RIB1* Is Important for Virulence of *Candida glabrata* in a Silkworm as a Model Host Organism

**FIGURE 5:**
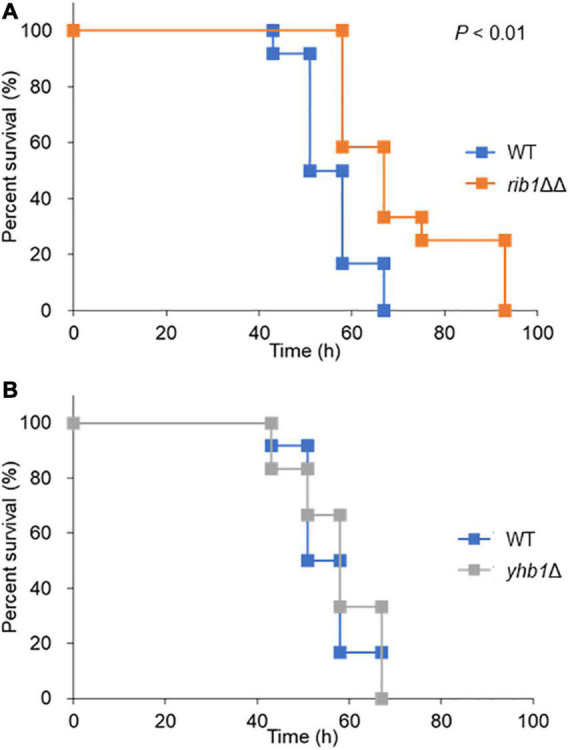
Infection assay using silkworm as a model host organism. **(A)** Survival rate of silkworm infected with *C. glabrata* strains. The *C. glabrata* strains (WT and *rib1*ΔΔ) were cultured in SC-Ura-His containing RF for 2 days. Resuspended yeast cells were injected to fifth-instar larvae using a needle. The larvae (*n* = 12) were maintained at 30°C, and survival rate was monitored for 4 days. Statistical significance was examined by Log-rank test using a statistical analysis software BellCurve. The *P* value for comparison between WT and *rib1*ΔΔ was lower than 0.01. **(B)** Survival rate of silkworm infected with *C. glabrata* strains. The *C. glabrata* strains (WT and *yhb1*Δ) were injected to fifth-instar larvae, and the survival rate was monitored for 4 days. There was no statistical significance between WT and *ybh1*Δ.

The silkworm has a similar innate immune system to that of mammals (Panthee et al., 2017); thus it is often used as a model organism for infection assays. The effect of *CgRIB1* on the virulence of *C. glabrata* was analyzed with an infection assay using silkworms. All silkworms died within 67 h after infection with the WT strain. On the other hand, silkworms infected with the *rib1*ΔΔ strain died at a significantly slower rate than those infected with the WT strain, indicating that *CgRIB1* is critical for the virulence of *C. glabrata* cells (Figure 5A). The survival rate of silkworms was not changed by the presence of the *YHB1* gene in *C. glabrata* (Figure 5B). These results suggest that *CgRIB1* is more important than *YHB1* for the virulence of *C. glabrata*.

## Discussion

We demonstrated that the CAGL0F04279g ORF functions as the *CgRIB1* gene encoding CgGCH2 and confers nitrosative stress resistance on *C. glabrata* cells. It was also shown that CgGCH2 is important for the proliferation of *C. glabrata* cells in macrophage-like cells *via* nitrosative stress resistance. The effect of NO on the virulence of *C. glabrata* has not been clarified previously, even though the lower NO production from macrophages activated by infection with *C. glabrata* was reported (Kasper et al., 2015). Thus, this is the first report to demonstrate the importance of nitrosative stress on infection by *C. glabrata*. Our findings provide new strategies for anti-bacterial and fungal therapies through the inhibition of CgGCH2 activity. Specific inhibitors of CgGCH2 should be considered as candidates for novel antifungals.

Furthermore, we found that *CgRIB1* is important for the virulence of *C. glabrata* by an infection assay using silkworms. We did not measure the RF content in silkworms; thus, we cannot exclude the possibility that the *rib1*ΔΔ strain had reduced its virulence because of RF auxotrophy inside silkworms. Previous studies have reported the importance of nitrosative stress in killing pathogens and protecting host organisms from pathogens during infection (Hromatka et al., 2005; Arai and Iiyama, 2013). Therefore, the increased survival rate of silkworms infected with the *rib1*ΔΔ strain could be caused by the decrease in nitrosative stress resistance of the *rib1*ΔΔ strain, at least partially.

Our DNA sequencing analysis suggested that the nucleotide sequences of two copies of *RIB1* were identical, from 509 bp upstream to 504 bp downstream of ORF. Although we did not determine the promoter region of each copy of *RIB1*, the GCH2 enzymatic activity in the cell-free extract from the *rib1*Δ strain was almost half of that from WT (Figure 2C), suggesting that the expression level of each *RIB1* copy is almost the same.

We showed that the nitrosative stress resistance of *C. glabrata* cells is dependent on the copy number of *CgRIB1* (Figure 3A). On the other hand, one copy of *CgRIB1* was enough to synthesize RF required for the growth of yeast cells (Figure 2A). These results raise the possibility that the function of CgGCH2 as an inducer for nitrosative stress resistance, not as an enzyme involved in RF synthesis, would be a selection pressure for evolution, as various situations including infection and symbiosis with other microbes generating NO can easily be imagined, even though the *CgRIB1* gene was unexpectedly duplicated during the construction of KUE100-1 strain from its host strain CBS138. The copy number variation of the *CgRIB1* gene might be a determinant for the virulence and pathogenicity of each strain in the same pathogenic microbe species.

The growth of the *yhb1*Δ strain on medium containing nitrite was better than that of *rib1*ΔΔ strain. This indicates that *CgRIB1* is more critical than *YHB1* for the nitrosative stress resistance of *C. glabrata*, at least under acidified nitrite conditions. The previous studies reported that the NO formation from nitrite under acidic conditions (pH 5.5) is extremely rapid (Opländer et al., 2012; Rayson et al., 2012). Linde et al. (2015) demonstrated that the NO donor *S*-nitrosoglutathione induced *YHB1* but not *CgRIB1* in *C. glabrata*. However, *CgRIB1* would be expressed for RF biosynthesis in the absence of RF without NO stress. Therefore, a constitutively active *CgRIB1*-dependent mechanism could alleviate the rapid and short-term NO stress derived from acidified nitrite more efficiently than *YHB1*, which might take a longer time to fully function. An inducible nitrosative stress-resistance system like *YHB1* is upregulated only after exposure to stress stimuli; it cannot function effectively at the beginning of stress exposure. In contrast, a constitutively active NO detoxifying mechanism such as *CgRIB1* is capable of modulating stress more rapidly. Although the constitutive activations of stress resistance mechanisms generally waste organismal resources, the activation of *CgRIB1* is required for the growth of yeast cells in the absence of RF thus that strategy is not a waste. A constitutive stress resistance mechanism that bifunctionally contributes to both stress response and basal growth, such as *CgRIB1*, would be a more effective and reasonable strategy for cell survival than an inducible strategy, especially under rapid stress conditions.

In contrast to the growth test on the solid medium containing nitrite, our infection assay with macrophages indicated that the *rib1*ΔΔ strain grew better than the *YHB1*-deleted strain at 24 h in the presence of RF (Figure 4C). The NO synthesis from the LPS-activated macrophage was much slower than that from acidified nitrite (Amura et al., 1998). The long-term nitrosative stress induced by the activated macrophage could be detoxified by the *YHB*-dependent resistance mechanism which was upregulated during exposure to NO for 24 h. However, the cell number of the *rib1*ΔΔ strain dropped at 48-h incubation, even though the *YHB1*-deficent strain still grew (Figure 4C). This implies that *CgRIB1* is more important than *YHB1* for the proliferation inside macrophage at this time point. Although the *CgRIB1*-dependent NO resistance mechanism should be involved in the proliferation in macrophage at 48 h incubation at least partly, the growth of the rib1ΔΔ strain was still worse than WT, thus other unknown mechanisms than NO resistance mediated by *CgRIB1* could contribute additionally. For example, it is possible that the RF concentration in macrophage at 48 h incubation is not enough to culture the RF auxotroph and then inhibits the growth of the *rib1*ΔΔ strain, since we did not determine the intramacrophage RF content. Whereas, *CgRIB1* might be induced more strongly than *YHB1* in macrophage at 48 h incubation, even though no researches to analyze the expression of *CgRIB1* inside macrophages have been reported.

We activated macrophage-like cells by treatment with LPS. Several pathogenic microorganisms, not only yeasts and fungi but also bacteria, co-infect. Polymicrobial infections of *Candida* species and bacteria are getting frequently in recent years (Klotz et al., 2007). Whereas, Coco et al. (2008) reported that the mixed infection of *C. glabrata* and *C. albicans* were important for the severe inflammation in denture wearers. These facts indicate that the coinfection of *C. glabrata* with other pathogenic microbes are not rare and the important situations to be examined. The infection of RAW264.7 cells activated by LPS with *C. glabrata* performed in this study can mimic such coinfection situations, even though the macrophage activation by infection with *C. glabrata* produces only a lower level of NO. Recently, it was reported that *C. glabrata* induced NO production from human osteoblasts (Muñoz-Duarte et al., 2016). Our infection analysis with the LPS-treated macrophage could also be a model system to examine the interaction with other types of cells.

## Conclusion

We identified the *RIB1* gene encoding GCH2 in *C. glabrata* (*CgRIB1*) by the RF auxotrophy test and the enzymatic analysis of the *CgRIB1* gene product. The growth test on the acidic medium containing nitrite indicated that *CgRIB1* was involved in nitrosative stress resistance in *C. glabrata*, which was dependent on its copy number. Our infection assay demonstrated that the *CgRIB1*-dependent nitrosative stress resistance is indispensable for the proliferation of yeast cells in macrophage-like cells and that *CgRIB1* plays a role for the virulence of *C. glabrata*. The findings in this study imply that the *CgRIB1*-dependent NO resistance system is a promising target for novel antifungals.

## Data Availability Statement

The raw data supporting the conclusions of this article will be made available by the authors, without undue reservation.

## Author Contributions

RN and HT conceived the study and designed the experiments. SS performed the experiments except for the infection assay with silkworms. SO and DH performed the infection assay with silkworms. RN, SS, DH, and HT analyzed the data. RN, DH, and HT wrote the manuscript. All authors read and approved the final manuscript.

## Conflict of Interest

The authors declare that the research was conducted in the absence of any commercial or financial relationships that could be construed as a potential conflict of interest.

## Publisher’s Note

All claims expressed in this article are solely those of the authors and do not necessarily represent those of their affiliated organizations, or those of the publisher, the editors and the reviewers. Any product that may be evaluated in this article, or claim that may be made by its manufacturer, is not guaranteed or endorsed by the publisher.
