# Supplementary material for: Identification and Functional Analysis of GTP Cyclohydrolase II in Candida glabrata in Response to Nitrosative Stress
Source: Front Microbiol. 2022 Mar 2;13:825121. doi: 10.3389/fmicb.2022.825121 (PMC8924521; doi:10.3389/fmicb.2022.825121)
Supplement: Supplementary file 1 [file Data_Sheet_1.PDF]

Table S1. List of primers used in this study.

Primer	Sequence (5’ to 3’)	Description
pUC19_IF_Fw	aggatccccgggtaccga	Construction for pUC18-ΔCAGL0F04279g-natNT2, and pUC18-ΔCAGL0F04279g-kanMX6
pUC19_IF_Rv	ctagagtcgacctgcaggca	
pUC19_dCgRIB1_IF1_Fw	gcaggtcgactctagctgtataactcactctatacgaacc	
pUC19_dCgRIB1_IF1_Rv	gtacccggggatccttttggactctaacacagataaattg	
dCgRIB1_IF2_vecFw	tacagtaaagatctttacacaga	
dCgRIB1_IF2_vecRv	ttgttgtattgaatttgaatttga	
dCgRIB1_IF2_insFw	aattcaatacaacaacgtacgctgcaggtcgac	
dCgRIB1_IF2_insRv	aagatctttactgtaatcgatgaattcgagctcgttt	
pUC19_dCgYHB1_IF1_Fw	gcaggtcgactctagtagaatgggctttttagtactaac	Construction for pUC18-YHB1-natNT2
pUC19_dCgYHB1_IF1_Rv	gcaggtcgactctagtagaatgggctttttagtactaac	
dCgYHB1_IF2_vecFw	gaaagatggatcatgatattctga	
dCgYHB1_IF2_vecRv	cttttgttgtttgttgtttcttggt	
dCgYHB1_IF2_insFw	aacaaacaacaaaagcgtacgctgcaggtcgac	
dCgYHB1_IF2_insRv	catgatccatctttcatcgatgaattcgagctcgttt	
CgRIB1_gateway_Fw	ggggacaagtttgtacaaaaaagcaggcttaatgtcccttccagttgtgc	Construction for pET53-CAGL0F04279g
CgRIB1_gateway_NoStop_Rv	ggggaccactttgtacaagaaagctgggtgtattgtttcttgctcttgaggg	
pUC19_dCgACT_IF1_Fw	aattcgtaatcatggtcatagctgtttcctgt	Construction for pUC18-CAGL0F04279g-*ACT1*
pUC19_dCgACT_IF1_Rv	cgagctcggtacccggggatc	
dCgACT_IF2_Fw	cgggtaccgagctcgatggattctggtatgttcga	
dCgACT_IF2_Rv	ccatgattacgaattttagaaacacttgtggtgaac	

Table S1. Continued.

pET53-CgRIB1-Fw	aacattaacatctagatggcacatcaccaccacc	Construction for pCU-PDC1-*ScRIB1*, and pCU-PDC1-CAGL0F04279g
pET53-CgRIB1-Rv	gggttgtgttctcgattacttttcgaactgcgggtgg	
dCgYHB1_Up500_Fw	gtagaatgggctttttagtact	Gene deletion
dCgYHB1_Down500_Fw	cgtattgttgaattttatgttg	
dCgRIB1_Up500_Fw	ctgtataactcactctatacgaaccattacac	Gene deletion, PCR analysis
dCgRIB1_Down500_Fw	tttggactctaacacagataaattgag	
clonNAT_R	cgatgtcctcgacggtcag	PCR analysis
kanmX4R	gtattgatgttggacgagtcgg	
CgRIB1_ORF522_ Rv	aagccttcagcttctcgctc	
CgRIB1_Fw_RT-PCR	ctgggcatcaagaacgtcag	qPCR
CgRIB1_Rv_RT-PCR	gtccagtggatcgggatcat	
CgACT1_Fw_RT-PCR	tccatcgtcggtagacca ag	
CgACT1_Rv_RT-PCR	aaacggccatggtgttatcg	

Table S2. List of yeast strains used in this study.

Strain	Genotype	Description
KUE100-1	*his3 ura3*	WT strain
*rib1*Δ	*his3 ura3 rib1::natNT2* pCU-PDC1 pRS313	*rib1* single disruptant
*rib1*ΔΔ	*his3 ura3 rib1::natNT2 rib1::kanMX6* pCU-PDC1 pRS313	*rib1* double disruptant
*yhb1*Δ	*his3 ura3 yhb1::natNT2* pCU-PDC1 pRS313	*yhb1* disruptant
RIB1OE	KUE100-1 pCU-PDC1-CAGL0F04279g pRS313	*CgRIB1* overexpression
*rib1*ΔΔ + *ScRIB1*	rib1ΔΔ pCU-PDC1-*ScRIB1* pRS313	Complemented strain
*rib1*ΔΔ + *CgRIB1*	rib1ΔΔ pCU-PDC1-CAGL0F04279g pRS313	Complemented strain

Table S3. List of plasmids used in this study.

Plasmid	Description
pUC18-ΔCAGL0F04279g-natNT2	Template to amplify the DNA fragment for CAGL0F04279g deletion by natNT2
pUC18-ΔCAGL0F04279g-kanMX6	Template to amplify the DNA fragment for CAGL0F04279g deletion by kanMX6
pUC18-*YHB1*-natNT2	Template to amplify the DNA fragment for *YHB1* deletion by natNT2
pUC18-CAGL0F04279g-*ACT1*	Template to draw the standard curve for quantitative PCR analysis
pCU-PDC1	Empty vector for *C. glabrata* harboring *URA3*
pRS313	Empty vector for *C. glabrata* harboring *HIS3*
pCU-PDC1-CAGL0F04279g	To express CAGL0F04279g under the control of the *PDC1* promoter
pCU-PDC1-*ScRIB1*	To express *ScRIB1* under the control of the PDC1 promoter
pET53-*CAGL0F04279g*	To express the recombinant protein encoded by CAGL0F04279g in *E. coli*
